# MiR-128-3p inhibits vascular smooth muscle cell proliferation and migration by repressing FOXO4/MMP9 signaling pathway

**DOI:** 10.1186/s10020-020-00242-7

**Published:** 2020-11-25

**Authors:** Chuan Qu, Xin Liu, Yan Guo, Yuhong Fo, Xiuhuan Chen, Jining Zhou, Bo Yang

**Affiliations:** Department of Cardiology, Renmin Hospital of Wuhan University, Wuhan University, Jiefang Road No.238, Wuhan, 430060 Hubei China

**Keywords:** MiR-128-3p, FOXO4, MMP9, Vascular smooth muscle cell, Atherosclerosis

## Abstract

**Background:**

MicroRNAs (miRNAs) have been identified as important participants in the development of atherosclerosis (AS). The present study explored the role of miR-128-3p in the dysfunction of vascular smooth muscle cells (VSMCs) and the underlying mechanism.

**Methods:**

Human VSMCs and ApoE knockout (ApoE^−/−^) C57BL/6J mice were used to establish AS cell and animal models, respectively. Expression levels of miR-128-3p, forkhead box O4 (FOXO4) and matrix metallopeptidase 9 (MMP9) were detected using qRT-PCR and Western blot, respectively. CCK-8, BrdU, and Transwell assays as well as flow cytometry analysis were performed to detect the proliferation, migration and apoptosis of VSMCs. Levels of inflammatory cytokines and lipids in human VSMCs, mice serum and mice VSMCs were also determined. The binding site between miR-128-3p and 3′UTR of FOXO4 was confirmed using luciferase reporter gene assay.

**Results:**

MiR-128-3p was found to be decreased in AS patient serum, ox-LDL-treated VSMCs, AS mice serum and VSMCs of AS mice. Transfection of miR-128-3p mimics suppressed the proliferation and migration of VSMCs, accompanied by the promoted apoptosis and the decreased levels of inflammatory cytokines. Further experiments confirmed the interaction between miR-128-3p and FOXO4. Augmentation of FOXO4 or MMP9 reversed the effects of miR-128-3p. Besides, miR-128-3p inhibited triglyceride (TG), total cholesterol (TC), low-density lipoprotein cholesterol (LDL-C) but increased high-density lipoprotein cholesterol (HDL-C) in the serum of AS mice.

**Conclusion:**

MiR-128-3p repressed the proliferation and migration of VSMCs through inhibiting the expressions of FOXO4 and MMP9.

## Introduction

Atherosclerosis (AS) is an inflammatory vascular disease, which contributes to the pathogenesis of a variety of cardiovascular diseases (CVD) (Gholipour et al. 2018). CVD remains the cause of about one third of mortality in the world (Moss and Ramji 2016). However, presently, the early diagnosis of AS is still difficult (Bejarano et al. 2018), and the treatment aims at repressing the levels of blood lipids, which has no direct effect on the formation of AS plaques (Orekhov and Ivanova 2016). The development of AS is elicited by inflammation and the dysfunction of vascular smooth muscle cells (VSMCs) (Paone et al. 2019). After vascular injury, VSMCs undergo phenotype-switching, followed by the release of inflammatory factors and the abnormal proliferation and migration, contributing to the formation of AS plaques; receptors such as LOX-1 on the cell membrane of VSMCs, activated by inflammatory factors such as TNF-α, will further promote the inflammatory responses through signaling pathways such as NF-κB, increase the expressions of cell adhesion factors, and thus further accelerate AS development (Byon et al. 2015; Lim and Park 2014; Jang et al. 2017). Therefore, modulating the inflammatory responses and the phenotypes of VSMCs is a potential strategy to repress the development of AS.

MicroRNAs (miRNAs) are non-coding RNAs with a length of 19–25nt. Accumulating researches suggest that miRNAs may be potential diagnostic and therapeutic targets for AS (Laffont and Rayner 2017). For example, miR-181b is found to be significantly increased in AS plaques, and in AS animal models established using ApoE and LDLR knockout mice, miR-181b enhances the stability of AS plaques by inhibiting target genes such as TIMP-3 and Elastin (Gregoli et al. 2017). MiR-128-3p is a tumor suppressor in a variety of tumors. For instance, in breast cancer, miR-128-3p triggers cell cycle arrest in cancer cells by repressing LIMK1 (Zhao et al. 2019a). In recent years, miR-128-3p has also been found to exert a protective role in CVD. It has been found that oxidized low density lipoprotein (OX-LDL)-treated RAW264.7 cells present remarkable reduction of miR-128-3p expression in a time and dose-dependent manner; while after the transfection of miR-128-3p mimics, it was found that both apoptosis and inflammatory responses were suppressed in RAW264.7 cells (Chen et al. 2018). This suggests that miR-128-3p can repress ox-LDL-induced inflammation and oxidative stress in macrophages, and inhibit the progression of AS.

Forkhead box protein O4 (FOXO4) is a member of the forkhead box O protein family. In cancer biology, FOXO4 is often considered to exert tumor-suppressive effects (Wang et al. 2016). An increasing number of studies have found that FOXO4 also has a regulatory role in AS development. For example, adiponectin is reported to exert protective effects on endothelial cells through inhibiting FOXO4 and inactivating NLRP3 inflammasome (Zhang et al. 2019). In ox-LDL-treated VSMCs, aberrant over-expression of lncRNA LINC00341 indirectly promotes the expression of FOXO4 by down-regulating miR-214, and thus promote the proliferation and migration of VSMCs (Liu et al. 2019a). Matrix metalloproteinase 9 (MMP9) is one of the downstream proteins regulated by FOXO4 (Li et al. (2007)). Previous studies have shown that MMP9 expression is significantly increased in human AS plaques (Balzan and Lubrano 2018); in an animal model of AS established using rabbits, irbesartan reduces AS lesion area by inhibiting the activation of NF-κB and repressing the expressions of COX-2 and MMP9 (Li et al. 2018).

In the present study, we established AS cell models and animal models using human VSMCs and ApoE knockout mice, to explore the roles of miR-128-3p, FOXO4 and MMP9 in the dysfunction of VSMCs and their regulatory relationship. It was demonstrated that miR-128-3p, functioning as a protective factor in AS development, repressed the proliferation and migration of VSMCs through inhibiting FOXO4/MMP9 axis.

## Methods and materials

### Clinical samples

We collected serum samples derived from 48 patients who were diagnosed with carotid atherosclerotic plaque in Renmin Hospital of Wuhan University from February 2018 to April 2019; meanwhile serum samples derived from 48 healthy volunteers were collected and set as controls. All patients and volunteers signed the written informed consent, and this study protocol had been approved by the Ethics Committee of Renmin Hospital of Wuhan University. In AS group, the age was 67.4 ± 13.4. 27 of the AS patients were male and 21 of the AS patients were female. The age of healthy volunteers was 68.0 ± 12. The gender composition of healthy volunteers was as same as AS group.

The inclusion and exclusion criteria of AS patients were as follows: Inclusion criteria: (1) abnormal lipid metabolism; (2) over 40 years old; (3) arteriography revealed a narrowing of the lumen caused by AS; (4) the local intraarterial membrane thickness > 1.5 mm. Exclusion criteria: (1) patients with autoimmune diseases or endocrine and metabolic diseases, including multiple arteritis, rheumatoid arthritis, thyroid dysfunction, adrenocortical dysfunction, diabetes etc.; (2) history of stroke, acute myocardial infarction, etc.; (3) congenital artery stenosis; (4) severe hepatic and renal insufficiency; (5) patients with malignant tumor or who was receiving anti-tumor treatment such as radiotherapy and chemotherapy; (6) pregnant or lactating women.

The inclusion criteria and exclusion criteria of healthy volunteers were as follows: Inclusion criteria: Physical examination, including examination of blood lipids, blood pressure, blood glucose, electrocardiogram, and markers of myocardial injury, etc., revealed no abnormality. Exclusion criteria: (1) diagnosed with AS; (2) history of cardiac arrest, myocardial infarction, or PCI; (3) pregnant or lactating women.

### Cell culture and transfection

Human VSMCs were purchased from the Chinese Academy of Sciences (Shanghai, China). The cells were maintained in Dulbecco’s Modified Eagle’s Medium (DMEM) (Thermo Fisher, HyClone, UT, USA) containing 10% fetal bovine serum (FBS) (Thermo Fisher Scientific, MA, USA) in 5% CO_2_ at 37 ℃. The medium was changed every 2 days, and the cells were passaged every 4–5 days. The cells in logarithmic phase were harvested for subsequent experiments.

After being trypsinized and resuspended using DMEM, VSMCs were seeded in 6-well plates at a density of 5 × 10^6^ cells / well, and when the cell confluency reached 70%, the cells were transfected with pcDNA3.1-FOXO4, pcDNA3.1-MMP9, miR-128-3p mimics, miR-128-3p inhibitors and their corresponding negative controls, respectively. All the above plasmids, miRNAs were constructed or synthesized by GenePharma (Shanghai, China). The transfection was performed with Lipofectamine™ 2000 reagent (Invitrogen, Carlsbad, CA, USA). Subsequently, the cells were cultured at 37 °C in 5% CO_2_. After 24 h of the transfection, total RNAs was extracted from the cells and the transfection efficiency was detected using quantitative real-time polymerase chain reaction (qRT-PCR).

VSMCs were treated with 0, 25, 50, 75, and 100 mg/L ox-LDL for 24 h or 100 mg/L ox-LDL for 0, 6, 12, 18, and 24 h to establish cell models of AS.

### Establishment of mouse model with AS

40 male C57BL/6J mice aged 6–8 weeks were purchased from Center for Animal Experiment of Wuhan University, of which 30 were apolipoprotein E knockout mice (ApoE^−/−^ Mice), and 10 were wild-type (WT) C57BL/6J mice. Of the WT mice, 5 WT mice were fed with normal diet (ND) and 5 were fed with high-fat diet (containing 20% lard oil and 0.25% cholesterol, purchased from Keao Xieli Feed Co., LTD., Beijing, China) for 8 weeks. Of the ApoE^−/−^mice, 5 were fed with ND and 25 were fed with HFD to establish AS models. All the mice had free access to food and water and were housed with humidity of 50%–60% at 18–22 °C. Manual-controlled room lighting was used to maintain a 12 h light/12 h dark cycle. 20 of these HFD ApoE^−/−^ mice were divided into four groups, 5 mice in each group: mimics NC group, miR-128-3p mimics group, inhibitors NC group, and miR-128-3p inhibitors group. After these four groups of mice were acclimated to the environment for 1 week, miR-128-3p mimics, inhibitors and their corresponding negative controls were dissolved in 0.2 mL of saline at a dose of 40 mg/kg/days and injected into the mice of corresponding groups through the caudal vein, respectively, every 2 weeks. 8 weeks later, the mice were sacrificed, and the chest was opened along the midline. The carotid artery was observed under stereomicroscope to confirm the AS. The carotid artery was carefully excised. The vascular outer layer was dissected, and the aorta was vertically cut with an ophthalmic scissors, and the intimal layer was scraped off with cotton swabs, and the vascular smooth muscle layer was obtained. The carotid smooth muscle cells of the mice were isolated and the serum samples were collected. The protocols of the animal experiments had been approved by the Animal Experiment Ethics Committee of Renmin Hospital of Wuhan University.

## qRT-PCR

Total RNAs from human VSMCs, mice carotid artery smooth muscle cells and serum were extracted using TRIzol reagent and serum RNA extraction kit (Invitrogen, Carlsbad, CA, USA), For quantifying FOXO4 and MMP9, 1 μg of the extracted total RNA was reversely transcribed into cDNA with PrimeScript-RT Kit (Takara, Kusatsu, Japan) after the purity was determined. For quantifying miR-128-3p, cDNA was generated via a miRNA reverse transcription kit (Origene, Rockville, MD, USA) according to the manufacturer’s instruction. With cDNA as the template, PCR amplification was performed with SYBR Green Premix Ex Taq II (TaKaRa, Dalian, China) on the Applied Biosystems™ 7500 Fast Dx Real-Time PCR Instrument (Applied Biosystems, Foster City, CA, USA). The primers used in this study were shown in Table 1. Relative expression was calculated with 2^−ΔΔt^ method.

**Table 1:** Primer sequences used in this PCR

Name	Primer sequences
MiR-128-3p	Forward: 5′-GGTCAGTGAACCGGTC-3′
	Reverse: 5′-GTGCAGGGTCCGAGGT-3′
FOXO4	Forward: 5′-CTTTCTGAAGACTGGCAGGAATGTG-3′
	Reverse: 5′-GATCTAGGTCTATGATCGCGGCAG-3′
MMP9	Forward: 5′-TTCCAAACCTTTGAGGGCGA-3′
	Reverse: 5′-CAAAGGCGTCGTCAATCACC-3′
U6	Forward: 5′-CTCGCTTCGGCAGCACA-3′
	Reverse: 5′-AACGCTTCACGAATTTGCGT-3′
β-Actin	Forward: 5′-CCTGGCACCCAGCACAAT-3′
	Reverse: 5′-TGCCGTAGGTGTCCCTTTG-3′

## Western blot

Cells were washed 3 times using pre-cooled PBS. Following that, RIPA lysis buffer (Beyotime Biotechnology, Shanghai, China) containing protease inhibitor was added and mixed thoroughly. The cells were placed on ice for 30 min. After centrifuging at 12,000 g for 10 min at 4 °C, the supernatant was collected and the protein concentration was determined using BCA protein detection kit (Beyotime Biotechnology, Shanghai, China). After that, the protein sample was subjected to electrophoresis and electrotransfered to PVDF membrane (Millipore, Bedford, MA, USA). After being blocked with 5% skim milk for 1 h at room temperature, the primary antibodies (anti-FOXO4 antibody, rabbit anti-human polyclonal antibody, 1:1000, ab63254; or anti-MMP9 antibody, rabbit anti-human polyclonal antibody, 1:1000, ab38898) were used to incubate the membranes overnight in a shaker at 4 ℃, and the membranes were then rinsed 3 times using TBST the next day. Next, the secondary antibody (goat anti-rabbit IgG, 1:2000, ab205718) was added and the membranes were incubated for 1 h at room temperature in a shaker. After the membranes being washed 3 times using TBST, the protein bands on the membrane were visualized using hypersensitive ECL (Biossci Biotechnology Co, Ltd., Wuhan, China). All antibodies were purchased from Abcam (Shanghai, China). The intensity of each band was analyzed by Image J software.

## CCK-8 assay

VSMCs in logarithmic phase were harvested, trypsinized and centrifuged. After being resuspended and adjusted to a cell density of 1 × 10^5^ cells/mL using DMEM, the cells were seeded into 96-well culture plates (100 μL / well) and then cultured at 37 ℃ in 5% CO_2_. After 0, 24, 48, 72, and 96 h of culture, 10 μL of CCK-8 solution (Beyotime, Shanghai, China) was added into each wells, respectively and the incubation was continued for 1 h. The absorbance of each well at 450 nm wavelength was measured using a microplate reader (ThermoFisher, Waltham, MA, USA). In this assay, each well had three biological replicates and three technical replicates.

## BrdU assay

VSMCs in logarithmic phase were prepared into single-cell suspensions and seeded into 24-well plates (1 × 10^5^ cells/well). 12 h later, BrdU labeled reagents (Beyotime, Shanghai, China) were added into the wells and the cells were incubated for 12 h. Then the cells were incubated with anti-BrdU antibody and DAPI staining solution (Beyotime, Shanghai, China), respectively. Next, three fields of view under the microscope were randomly selected for cell counting. Cell proliferation rate = BrdU-positive cells / DAPI-positive cells. The average of the cell proliferation rates in three fields was adopted as the final cell proliferation rate.

## Flow cytometry

AnnexinV-FITC/PI apoptosis detection kit (Yeasen Biotech Co., Ltd., Shanghai, China) was employed to detect the apoptosis of VSMCs. In brief, 1 × 10^6^ cells in each group were harvested, and resuspended in 100 μL of 1 × binding buffer, followed by the addition of 5 μL of Annexin V-FITC staining solution and 5 μL of PI staining solution to the cells, mixed thoroughly and incubated in dark at room temperature for 15 min. The apoptosis rate was detected by flow cytometry within 1 h.

## Transwell assay

The cells in each group were trypsinized, and the cells were resuspended using DMEM without serum to adjust the cell density to 1 × 10^5^ cells/mL, and 200 μL of cell suspension was added to the upper compartment of the Transwell chamber (Corning, Beijing, China), meanwhile 500 μL of DMEM containing 10% FBS was added to the bottom compartment and the cells were cultured at 37 ℃, 5% CO_2_ for 48 h. After the chamber was removed, the cells on the bottom of the membrane were fixed with 4% paraformaldehyde for 15 min, stained with 0.1% crystal violet solution for 15 min, and the remaining crystal violet solution was washed off using PBS, and the cells in the upper chamber were cleaned using a cotton swab. Five fields of view on the membrane under the microscope were used to count the number of cells and the average was calculated to indicate the migration ability of VSMCs.

### Dual-luciferase reporter gene assay

The genomic DNA was extracted from human VSMCs. Then the FOXO4 3′UTR sequence containing the putative binding site for miR-128-3p was amplified. GeneArt™ site-directed Mutagenesis PLUS System (ThermoFisher, Waltham, MA, USA) was used to mutated the above sequences. After gel electrophoresis of the amplified products, the target sequences were collected, and the above fragments were inserted into the pmirGLO dual-Luciferase miRNA Target expression vector (Promega, Madison, WI, USA) to construct the reporter vectors: FOXO4 wild type (WT) and FOXO4 mutant (MUT). After the sequence of the reporter vectors were validated by Sangon Biotech (Shanghai, China) with sequencing, the reporter vectors were transfected into the cells, respectively, together with miR-128 mimic or mimics-NC. The medium was discarded after 48 h. The cells were washed using PBS and then cell lysis buffer was added to the wells to lyse the cells. After being centrifuged at 3000×*g* for 5 min, the supernatant was harvested for the detection of luciferase activity with dual-luciferase reporter assay system (Promega, Madison, WI, USA).

### Determination of inflammatory factors

The levels of TNF-α, IL-1β and IL-6 in the cell culture supernatant or mice serum were detected using enzyme-linked immunosorbent assay (ELISA) kits (Multisciences, Hangzhou, China) according to the manufacturer’s instructions.

### Determination of lipid levels

The levels of total cholesterol (TC), triglyceride (TG), low-density lipoprotein cholesterol (LDL-C) and high-density lipoprotein cholesterol (HDL-C) in mice serum were detected using corresponding detection kits (Jiancheng Bioengineering Institute, Nanjing, China) according to the manufacturer’s instructions.

### Statistical analysis

All data in this study were processed using SPSS 20.0 statistical analysis software (SPSS Inc., Chicago, IL, USA). The measurement data were expressed as "mean ± standard deviation" (x ± s). The comparison between two groups was performed using independent sample *t*-test. The comparison between multiple groups was analyzed with one-way ANOVA analysis. *p* < 0.05 signified statistical significance.

## Results

### miR-128-3p expression was abnormally down-regulated during AS progression

**Fig. 1:**
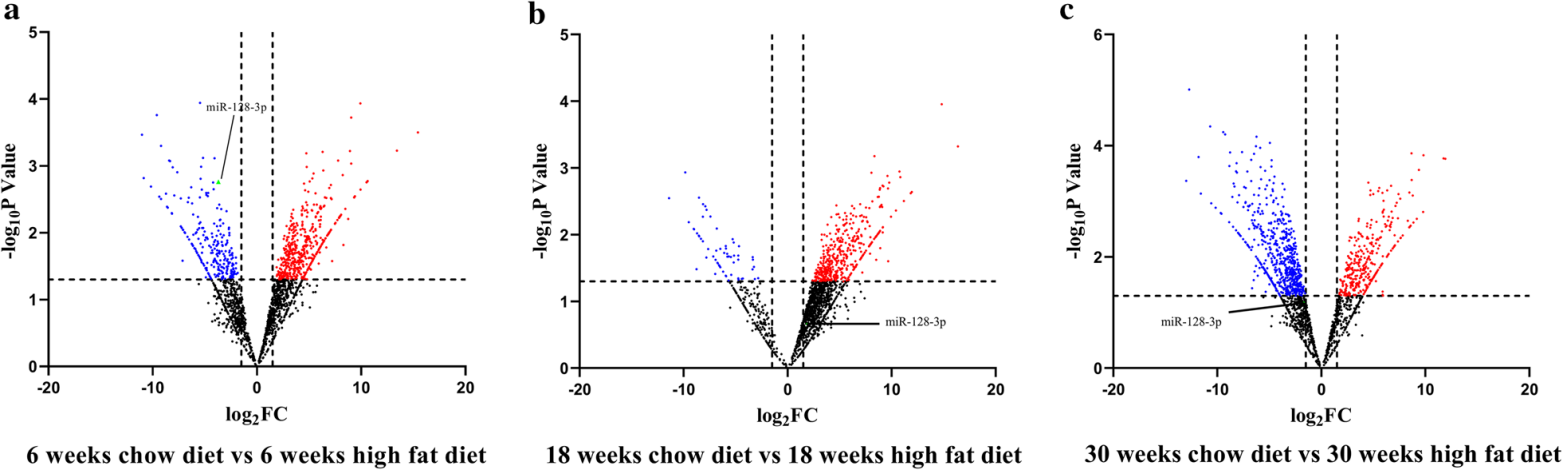
Discovery of miR-128-3p via GEO dataset. **a**–**c** miRNA expression profile (GSE89858) in AS lesions in the ascending aorta of Apobtm2Sgy/Ldltm1Her double knockout mice fed with high-fat diet for 6, 18 and 30 weeks (vs mice fed with normal diet for 6, 18 and 30 weeks)

**Fig. 2:**
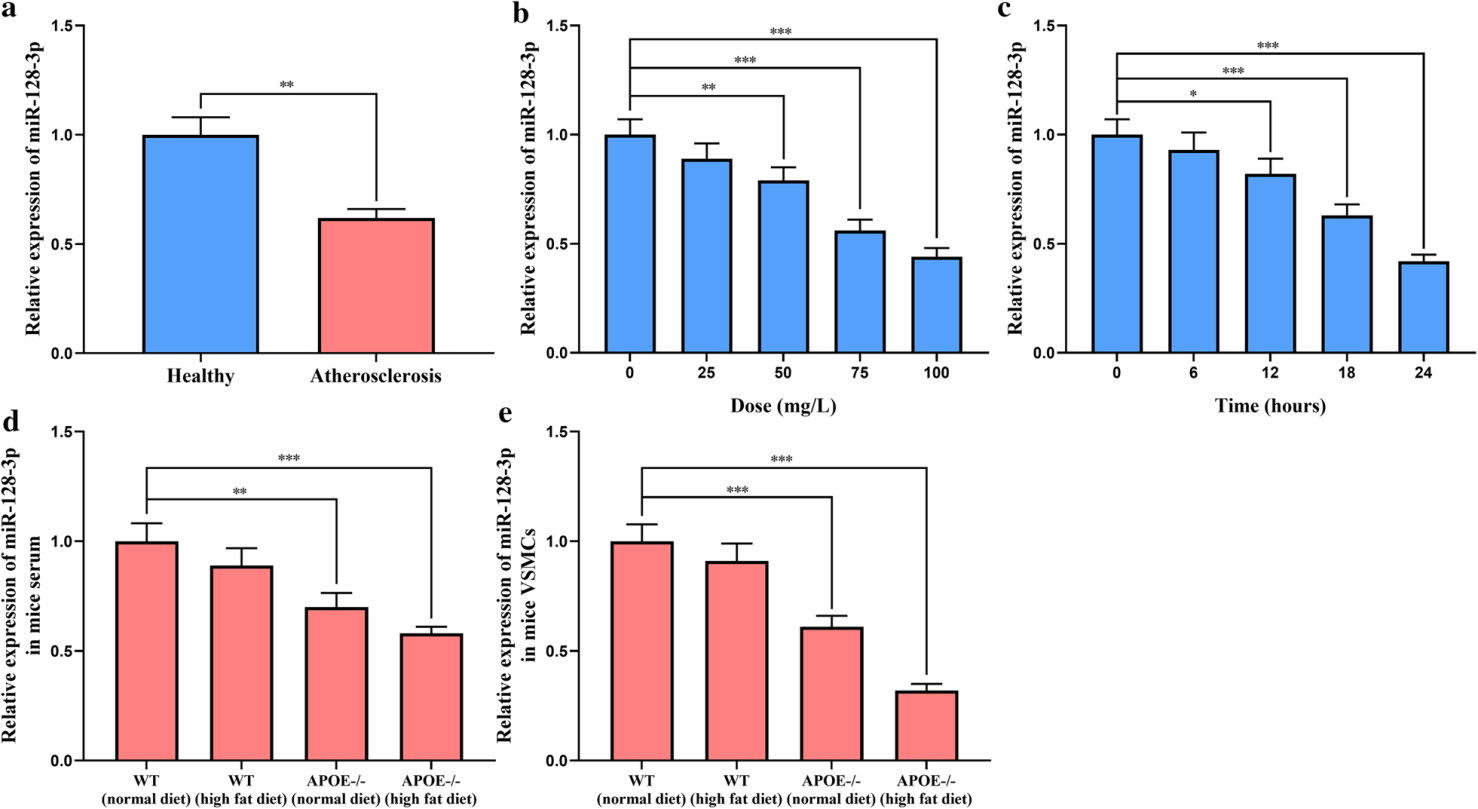
Expression of miR-128-3p during AS development. **a** qRT-PCR was used to detect the expression levels of miR-128-3p in serum of healthy subjects and AS patients. **b** qRT-PCR was used to detect the expression levels of miR-128-3p in VSMCs treated with different concentrations of ox-LDL for 24 h. **c** qRT-PCR was used to detect the expression levels of miR-128-3p in VSMCs after treatment with 100 mg/L ox-LDL for different times. **d**, **e** qRT-PCR was used to detect the expression levels of miR-128-3p in the serum (**d**) and carotid vascular smooth muscle (**e**) of the mice in different groups. *, **, *** represent *p* < 0.05, *p* < 0.01, *p* < 0.001, respectively

First of all, with bioinformatics analysis, it was found that in Apobtm2Sgy/Ldltm1Her double knockout mice, miR-128-3p expression was significantly reduced in AS lesions in the ascending aorta of mice fed with HFD compared with mice fed with ND after 6 weeks of feeding, based on the public miRNA expression profile dataset GSE89858, but no significant changes were found after 18 and 30 weeks of feeding (Fig. 1a–c). Next, to further investigate the role of miR-128-3p during AS progression, we examined its expression level using qRT-PCR. It was found that miR-128-3p expression was remarkably decreased in the serum of AS patients (Fig. 2a). In ox-LDL-treated VSMCs, the expression level of miR-128-3p was remarkably decreased with the increase of the concentration of ox-LDL and treatment time (Fig. 2b, c). Additionally, compared with wild-type mice fed with ND, the decrease of miR-128-3p expression was observed in the serum and carotid smooth muscle cells of ApoE^−/−^mice fed with HFD (Fig. 2d, e). The above results indicated that miR-128-3p expression was abnormally reduced in the development of AS.

### Effect of miR-128-3p on VSMCs

**Fig. 3:**
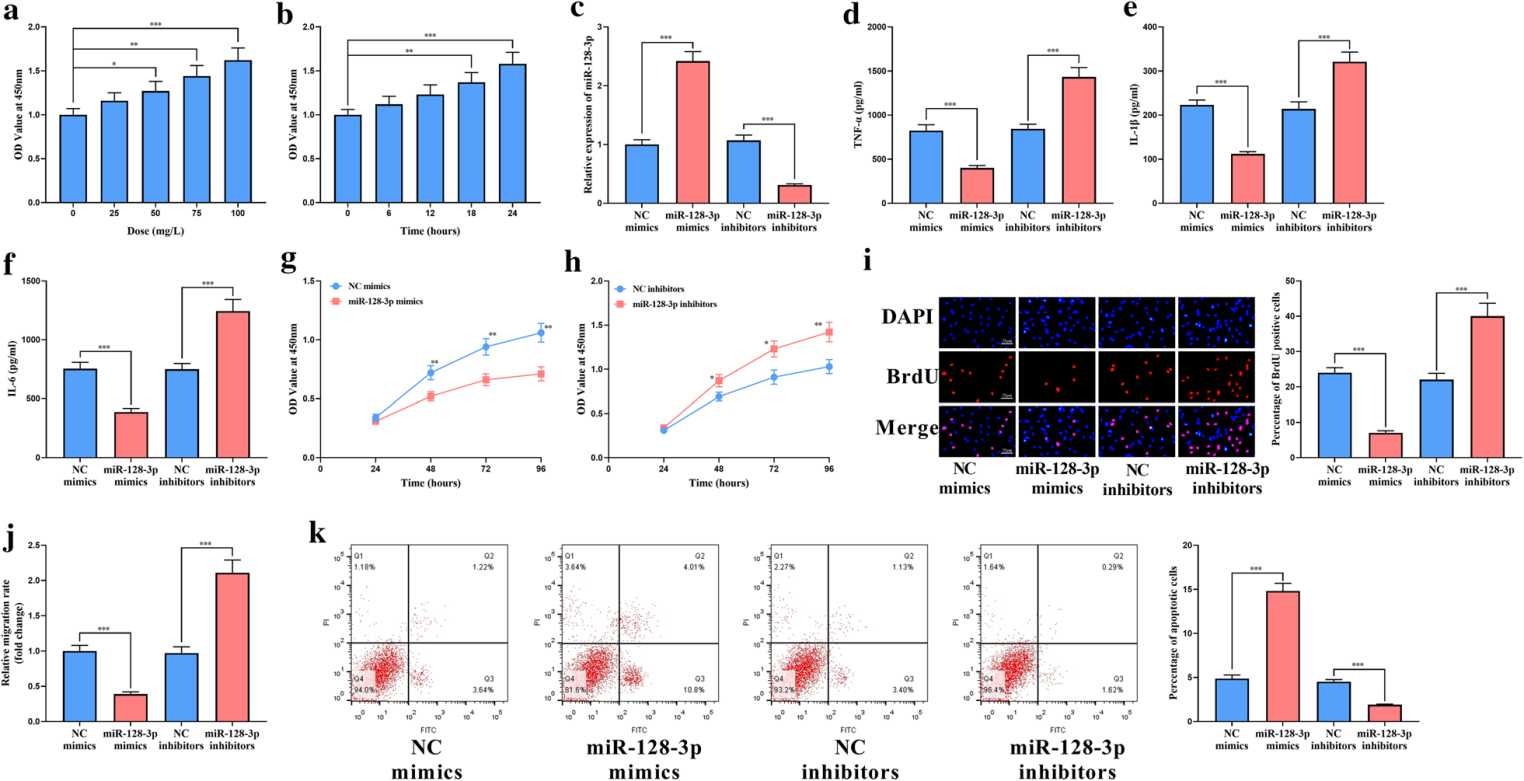
Effect of miR-128-3p on VSMCs. **a** CCK-8 assay was used to determine the viability of VSMCs treated with different concentrations of ox-LDL for 24 h. **b** CCK-8 assay was used to determine the viability of VSMCs treated with 100 mg/L ox-LDL for different stimulating times. **c** The transfection efficiency of miR-128-3p mimics and inhibitors was examined using qRT-PCR. The levels of inflammatory cytokines TNF-α (**d**), IL-1β (**e**) and IL-6 (**f**) in the supernatants of VSMCs were measured using ELISA. **g**, **h** VSMCs proliferation was measured using CCK-8 assay after the transfection. **i** VSMCs proliferation was measured using BrdU assay after the transfection. **j** VSMCs migration was examined using Transwell assay after the transfection. **k** VSMCs apoptosis was detected using flow cytometry after the transfection. *, **, *** represent *p* < 0.05, *p* < 0.01, *p* < 0.001, respectively

VSMCs were then treated with different concentrations of ox-LDL for different treatment times in vitro. We observed that, the viability of VSMCs was the highest when treated with 100 mg/L ox-LDL for 24 h (Fig. 3a, b). So this condition was used for the subsequent experiments. To investigate the function of miR-128-3p, we transfected miR-128-3p mimics or inhibitors into VSMCs to up-regulate or inhibit miR-128-3p, respectively (Fig. 3c). The levels of inflammatory factors in supernatants of VSMCs were determined using ELISA. The results showed that miR-128-3p over-expression markedly inhibited the release of TNF-α, IL-6 and IL-1β, while opposite results could be observed in the cells tranfected with miR-128-3p inhibitors (Fig. 3d–f). CCK-8 and BrdU assays suggested that miR-128-3p remarkably suppressed the abnormal proliferation of VSMCs, while miR-128-3p inhibitors significantly promoted the viability of VSMCs (Fig. 3g–i). In Transwell assay, it was found that the transfection of miR-128-3p mimics significantly repressed the migration of VSMCs, while the inhibition of miR-128-3p markedly promoted the migration of VSMCs (Fig. 3j). Moreover, through flow cytometry analysis, it was observed that miR-128-3p had a role in promoting apoptosis in VSMCs (Fig. 3k). These results indicated that miR-128-3p suppressed the dysfunction of VSMCs induced by ox-LDL.

### miR-128-3p targeted and regulated FOXO4/MMP9

**Fig. 4:**
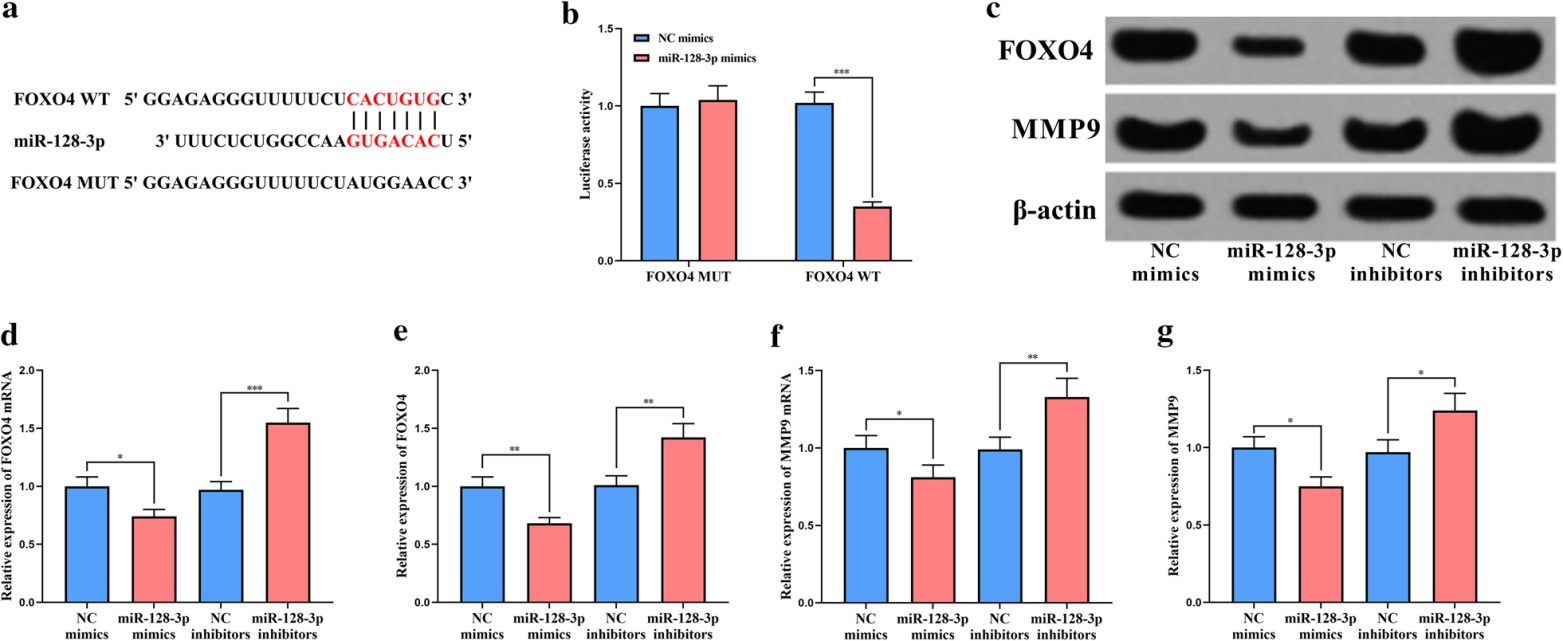
Effect of miR-128-3p on FOXO4 and MMP9. **a** Predicted binding site between miR-128-3p and the 3′UTR of FOXO4. **b** The binding relationship between miR-128-3p and FOXO4 3′UTR was verified by dual luciferase reporter gene assay. **c** Western blot was used to detect FOXO4 and MMP9 expressions after transfection. **d**–**g** The quantification of FOXO4 and MMP9 at the mRNA and protein levels in VSMCs after miR-128-3p was overexpressed or inhibited. *, **, *** represent *p* < 0.05, *p* < 0.01, *p* < 0.001, respectively

To further explore the downstream mechanism of miR-128-3p, we predicted the downstream target genes of miR-128-3p using TargetScan database and found that FOXO4 was one of the potential targets of miR-128-3p. Therefore, dual-luciferase reporter gene assay was performed, and it was observed that miR-128-3p mimics significantly reduced the luciferase activity of FOXO4 WT reporter, but there is no significant change presented in FOXO4 MUT group after miR-128-3p mimics were transfected (Fig. 4a, b). We further examined the expressions of FOXO4 and its downstream protein MMP9 using qRT-PCR and Western blot. It was found that the over-expression of miR-128-3p inhibited the expression levels of FOXO4 and MMP9 at both mRNA and protein levels (Fig. 4c–g).

### Restoration of FOXO4 or MMP9 reversed the effect of miR-128-3p on VSMCs

**Fig. 5:**
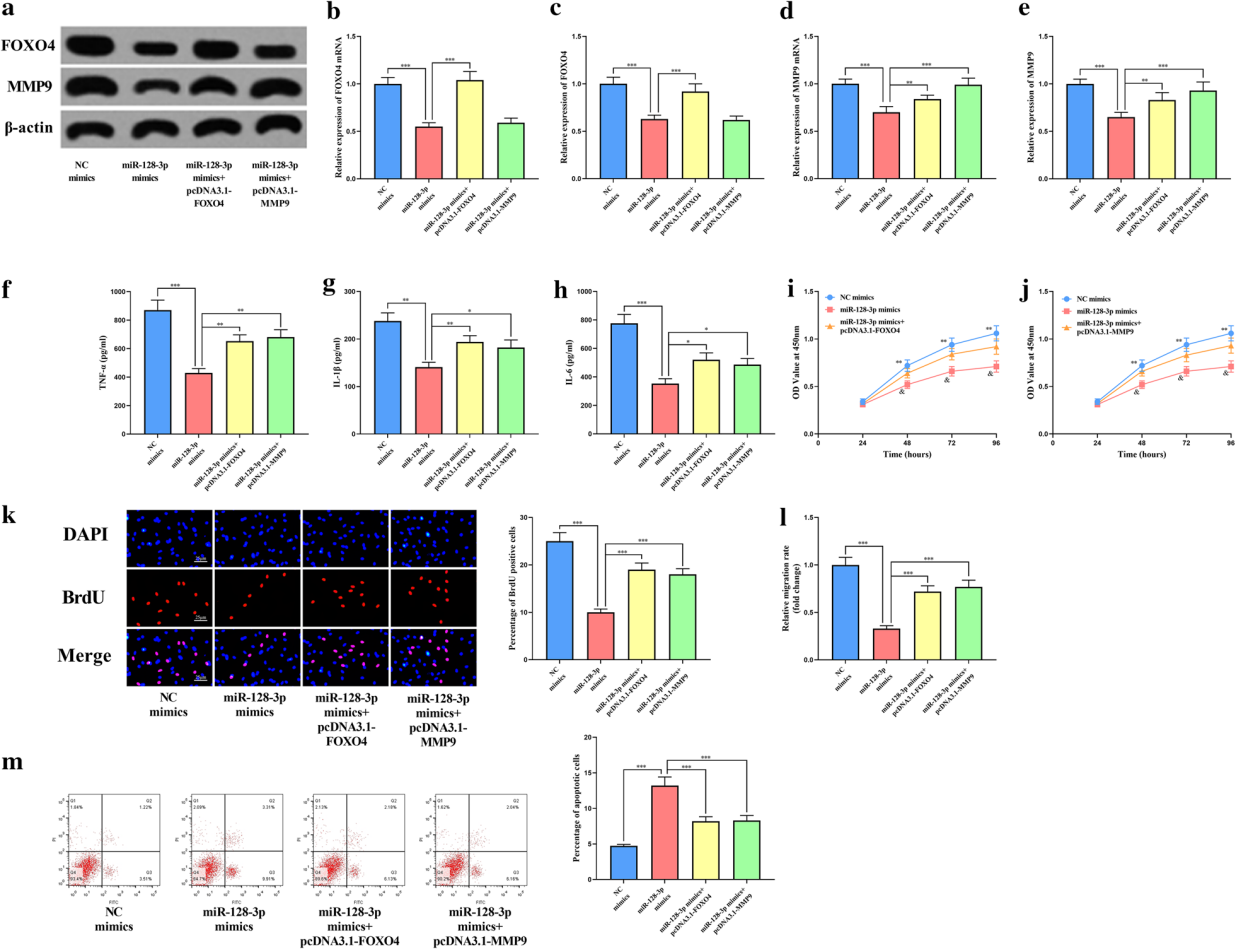
The effects of FOXO4 and MMP9 on miR-128-3p function. **a** The expressions of FOXO4 and MMP9 were detected by Western blot after transfection. **b**–**e** The quantification of FOXO4 and MMP9 at the mRNA and protein levels in VSMCs after transfection. The levels of inflammatory factors TNF-α (**f**), IL-1β (**g**) and IL-6 (**h**) in supernatants of VSMCs were examined by ELISA. **i**, **j** The proliferation of VSMCs was detected by CCK-8. **k** The proliferation of VSMCs was examined by BrdU assay. **l** The migration of VSMCs was examined by Transwell assay. **m** The apoptosis of VSMCs was examined by flow cytometry. *, **, *** represent *p* < 0.05, *p* < 0.01, *p* < 0.001, respectively

To further investigated the mechanism by which miR-128-3p functions, we co-transfected miR-128-3p mimics and pcDNA3.1-FOXO4 or pcDNA3.1-MMP9 into VSMCs. By qRT-PCR and Western blot, we verified that the expression level of FOXO4 or MMP9 was indeed restored after the transfection (Fig. 5a–e). The results of ELISA showed that over-expression of either FOXO4 or MMP9 could attenuate the inhibitory effects of miR-128-3p on ox-LDL-induced inflammatory responses in VSMCs (Fig. 5f–h), and the results of CCK-8 and BrdU assays confirmed that FOXO4 and MMP9 enhanced the proliferation of VSMCs (Fig. 5i–k). Through Transwell assay, it was observed that the inhibitory effect of miR-128-3p on VSMCs migration was also reversed by FOXO4 or MMP9 restoration (Fig. 5l). In addition, compared with that in the miR-128-3p mimics group, the apoptosis rate of VSMCs in miR-128-3p mimics + pcDNA3.1-FOXO4 group or miR-128-3p mimics + pcDNA3.1-MMP9 group was remarkably down-regulated (Fig. 5m).

### The biological functions of miR-128-3p in mice

**Fig. 6:**
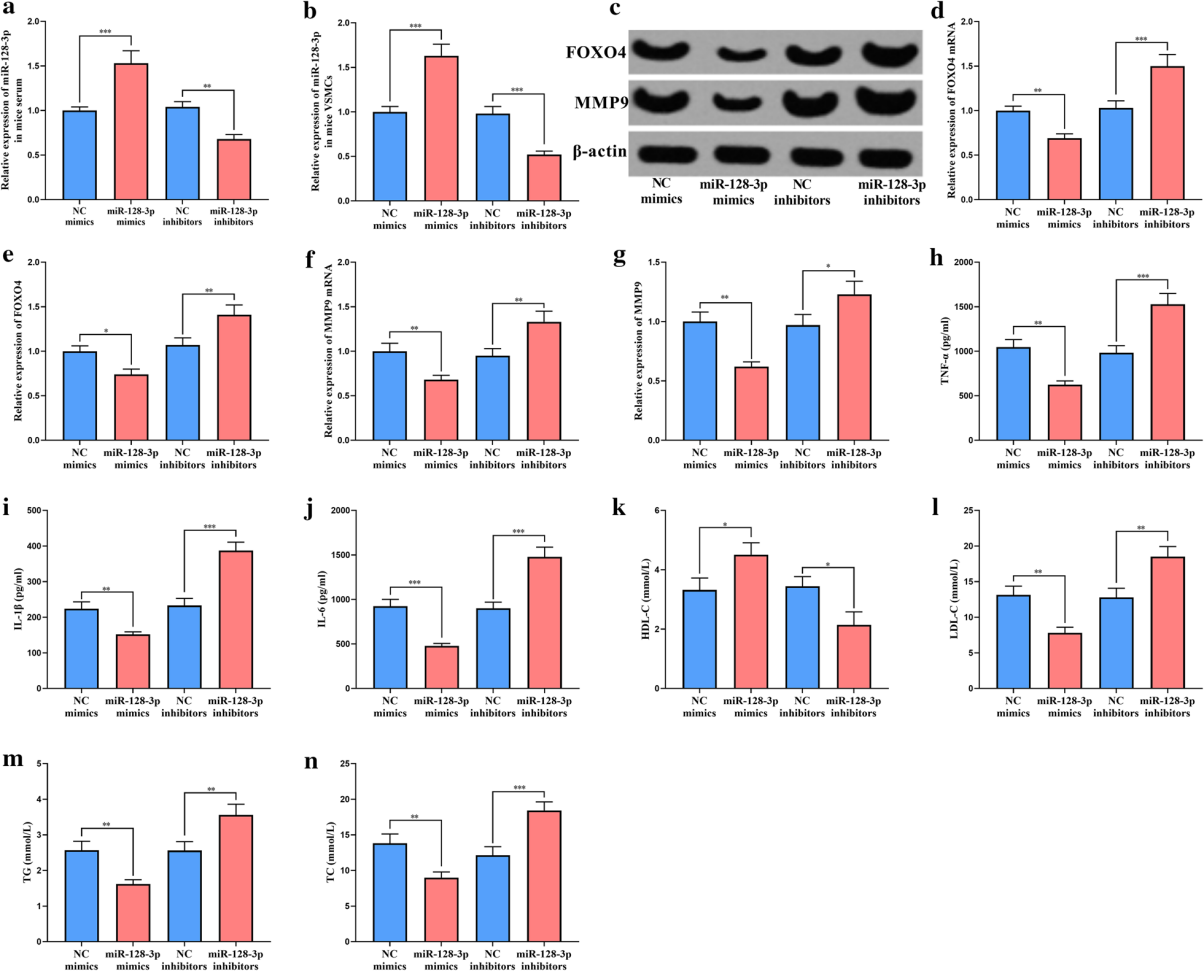
The functions of miR-128-3p in mice. qRT-PCR was used to detect the expression levels of miR-128-3p in the serum samples (**a**) and carotid artery smooth muscle cells **b** of the mice after injection. **c** The expressions of FOXO4 and MMP9 in mice carotid artery smooth muscle cells were detected by Western blot. **d**–**g** The quantification of FOXO4 and MMP9 at the mRNA and protein levels in mice carotid artery smooth muscle cells. Inflammatory cytokines TNF-α (**h**), IL-1β (**i**), and IL-6 (**j**) in the serum of the mice were determined by ELISA. **k**–**n** The levels of HDL-C, LDL-C, TG and TC in the serum of the mice. *, **, *** represent *p* < 0.05, *p* < 0.01, *p* < 0.001, respectively

We established animal models with AS by feeding ApoE^−/−^mice with HFD, followed by the injection of miR-128-3p mimics or inhibitors through the caudal vein to regulate miR-128-3p expressions in vivo. By qRT-PCR, we verified that miR-128-3p expression levels in mice serum and carotid smooth muscle cells were indeed changed after the injection (Fig. 6a, b). Western blot and qRT-PRC indicated that, the expression levels of FOXO4 and MMP9 at both mRNA and protein levels in mice carotid smooth muscle cells were also disturbed (Fig. 6c–g). The results of ELISA suggested that the injection of miR-128-3p mimics reduced the inflammatory responses in mice (Fig. 6h–j). In addition, it was also found that TG, TC, and LDL-C levels in the serum of the mice in miR-128-3p mimics group were markedly reduced, while HDL-C level was significantly increased (Fig. 6k–n). Collectively, it was confirmed that miR-128-3p suppressed VSMCs dysfunction and regulated blood lipid levels through specific regulation of FOXO4/MMP9 axis.

## Discussion

In the present study, it was demonstrated that miR-128-3p was significantly down-regulated in the serum of AS patients, the serum of AS mice, the carotid artery smooth muscle cells of AS mice, and ox-LDL-treated VSMCs, and miR-128-3p could not only attenuate the inflammatory responses of VSMCs and inhibit their abnormal proliferation and migration, but also reduce the lipid levels in the serum of mice. In glioma, miR-128-3p expression is remarkably reduced, and it inhibits the proliferation and migration of glioma cells by specific inhibition of GREM1 (Fu et al. (2018)). In esophageal squamous cell carcinoma, miR-128-3p, which is lowly expressed, inhibits the migration of cancer cells through inhibiting ZEB1 (Zhao et al. 2018). Reportedly, selenium markedly increases the expression of miR-128-3p and suppresses the MAPK signaling pathway, which in turn reduces the levels of proinflammatory factors including TNF-α and IL-1 in LPS-induced chicken myocardial inflammation (Liu et al. 2020a). These studies imply that miR-128-3p is an inhibitor for proliferation, migration and inflammation, which is consistent with the demonstrations in the present work. Notably, a recent research reports that miR-128-3p is significantly down-modulated in VSMCs treated with PDGF-BB or hypoxia, and the aortic samples of ApoE^−/−^ mice fed with Western diet; overexpression of miR-128-3p decreases VSMCs proliferation, migration and helps VSMCs maintain a contractile phenotype (Farina et al. 2020). The results are consistent with the present study.

**Fig. 7:**
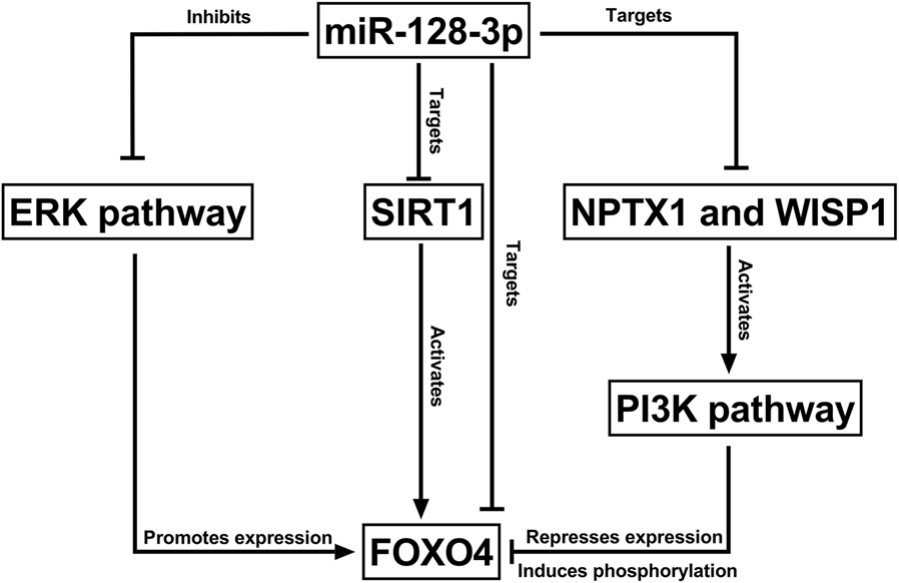
Potential indirect regulatory functions of miR-128-3p on FOXO4. MiR-128-3p may regulate FOXO4 expression via ERK pathway, SIRT1 and PI3K pathway

MiRNAs often exert their function by inhibiting the post-transcriptional translation of their downstream target genes. In this work, it was also confirmed that miR-128-3p could suppress FOXO4 expression, and the functions of miR-128-3p in regulating the phenotypes of VSMCs were mediated by FOXO4. In cancer research, FOXO4 is often considered to have a role in impeding cell proliferation (Liu et al. 2011; Chen et al. 2013). However, in the present study, it was observed that the FOXO4 had a role in enhancing the proliferation and migration of VSMCs, which was different from its role in cancer cells. FOXO4 expression is significantly higher in damaged arteries, and lncRNA XR007793 facilitates the proliferation and migration of VSMCs by indirectly up-regulating FOXO4 (Wu et al. 2018). Under the stimulation of ox-LDL, lncRNA LINC00341 expression is increased in VSMCs, which enhances the proliferation and migration of VSMCs by promoting the expression of FOXO4 (Liu et al. 2019a). The above demonstrations are consistent with our work, and collectively, we suppose that inhibiting FOXO4 will probably block the progression of AS via repressing the dysfunction of VSMCs. Importantly, miR-128-3p may not only regulate FOXO4 expression through binding its 3′UTR. MiR-128-3p is reported to regulate various pathways / genes, which can probably regulate FOXO4 expression, including Erk signaling, SIRT1, PI3K/AKT pathway and so on (Liu et al. 2020b, 2019b; Wang et al. 2014; Zhao et al. 2019b; Kobayashi et al. 2005; Huo et al. 2019; Chen and Li 2020; Gong et al. 2019; Chang et al. 2013) (Fig. 7). In VSMCs, whether miR-128-3p indirectly modulates FOXO4 expression through these mechanisms needs more investigations in the future.

MMP9 is an important regulator in inflammatory response. Leukocytes recruited by the inflammatory responses produce a variety of cytokines and chemokines that facilitate the release of MMP9, which also has a role in promoting the activation of inflammatory factors such as IL-1β, forming a positive feedback (Li et al. 2007; Deleon-Pennell et al. 2015). MMP9 is often increased when AS occurs, and the level of MMP9/NGAL complex is strongly related to the vulnerability of AS plaques (Eilenberg et al. 2017; Wu et al. 2019). It is reported that the expression of MMP9 is positively regulated by FOXO4 (Li et al. 2007), which is consistent with our findings. We suppose that, at least partly, miR-128-3p and FOXO4 exert their biological functions through regulating MMP9 in AS development.

Hyperlipidemia and dysregulation of lipoproteins are important factors in AS pathogenesis. In hyperlipidemia, circulating platelets are activated, accompanied by increased platelet aggregation, platelet-leukocyte aggregate formation, and platelet-induced superoxide anion production; furthermore, ox-LDL induces monocytes’ adhesion to endothelium, migration and proliferation of smooth muscle cells. These biological events contributes to AS progression (Siegel-Axel et al. 2008; Bühler et al. 1991). Interestingly, in the present work, we found that injection of miR-128-3p mimics significantly reduced LDL-C, TG, TC but elevated HDL-C in the plasma of mice, which suggested that miR-128-3p may also repress AS progression via regulating lipid metabolism, but the detailed mechanism needs further clarification.

## Conclusion

In conclusion, with in vitro and in vivo models, this work indicates that miR-128-3p/FOXO4/MMP9 axis regulates AS progression via modulating the inflammatory response, proliferation and migration of VSMCs. Our research further elucidates the molecular mechanism of AS development, and provides novel insights into prevention and treatment of CVD.

## Data Availability

The data used to support the findings of this study are available from the corresponding author upon request.
